# Enhancing participation in research for Asian American, Native Hawaiian, and Pacific Islander Populations: An update on the CARE Registry

**DOI:** 10.1002/alz.091617

**Published:** 2025-01-09

**Authors:** Van Ta Park, Janice Y. Tsoh, Oanh L. Meyer, Bora Nam, Marian Tzuang, Quyen Vuong, Ladson Hinton, Dolores Gallagher‐Thompson, Alka M. Kanaya, Daren Huang, Gabriel Fara‐On, Joshua D Grill

**Affiliations:** ^1^ Asian American Research Center on Health (ARCH), University of California San Francisco, San Francisco, CA USA; ^2^ University of California San Francisco School of Nursing, San Francisco, CA USA; ^3^ University of California San Francisco School of Medicine, San Francisco, CA USA; ^4^ University of California, Davis School of Medicine, Sacramento, CA USA; ^5^ International Children Assistance Network, Milpitas, CA USA; ^6^ Stanford University School of Medicine, Stanford, CA USA; ^7^ Institute for Memory Impairments and Neurological Disorders, University of California, Irvine, Irvine, CA USA

## Abstract

**Background:**

Asian American, Native Hawaiian, and Pacific Islander (AANHPI) populations are underrepresented in Alzheimer’s disease and related dementias (ADRD) research, despite being the fastest growing racial group in the United States. The Collaborative Approach for AANHPI Research and Education (CARE) registry aims to create a sustainable research recruitment source to address this need.

**Method:**

Participants can enroll online, by phone, or in‐person by completing an enrollment survey in English, Chinese (Simplified/Traditional), Hindi, Korean, Samoan, or Vietnamese. Guided by a Community Advisory Board, CARE is strategically promoted as a registry that supports “health research across the lifespan,” focusing on ADRD, dementia caregiving, and aging‐related topics. CARE regularly holds Brain Trust Meetings to promote CARE in the research community, and researchers can request support for their study recruitment.

**Result:**

Between October 2020 and January 2024, 10,133 total AANHPI adults enrolled in the CARE registry. Participants’ mean age is 51.8 years (Standard Deviation (SD) = 17.9), including 2,450 middle‐aged (50‐64 years old) and 3,373 older (≥65 years old) participants. About 14% (n = 1,402) of the participants reported having ADRD symptoms. Among the 1,072 caregivers, 40.4% reported caring for someone with ADRD. Most participants who had self‐reported ADRD symptoms (96.4%) and caregivers (76.5%) were foreign‐born with 61.7% speaking limited English. The major cultural groups among ADRD caregivers were Chinese (33.2%), Vietnamese (26.5%), Korean (11.3%), Asian Indian (7.6%), and Japanese (8.8%). Of the 98 recruitment referral requests received, 64 (65.3%) were aging‐related, including 49 (50%) ADRD or ADRD‐caregiving studies. CARE shared data with 51 studies and over 5,500 CARE participants have been referred to at least one study from January 2021 to January 2024.

**Conclusion:**

CARE has successfully recruited over 10,000 AANHPI participants who are interested in health research participation. Participants from several AANHPI groups were limited (e.g., Filipino, Asian Indian, Native Hawaiians/Pacific Islanders), however, we anticipate future growth following language expansions and targeted outreach efforts. The registry stands as a valuable recruitment source and fosters meaningful and necessary engagement of AANHPI representation in ADRD‐related research.

